# Molecular Investigation of the p28 Gene of *Ehrlichia canis* in Infected Dogs From Ho Chi Minh City, Vietnam

**DOI:** 10.1155/vmi/8884821

**Published:** 2025-10-06

**Authors:** Nguyen Thi Lieu Kieu, Chien Tran Phuoc Nguyen, Tran Thi Thao, Tran Ngoc Bich

**Affiliations:** ^1^Interdisciplinary Graduate Program in Veterinary Therapeutics and Pathology, Faculty of Veterinary Medicine, College of Agriculture, Can Tho University, Campus II, 3/2 Street, Ninh Kieu Wards, Can Tho, Vietnam; ^2^Faculty of Veterinary Medicine, College of Agriculture, Can Tho University, Campus II, 3/2 Street, Ninh Kieu Wards, Can Tho, Vietnam

**Keywords:** canine monocytic ehrlichiosis, *Ehrlichia canis*, entropy analysis, p28 gene, phylogenetics, Vietnam

## Abstract

*Ehrlichia canis* (*E. canis*) is a tick-borne, obligate intracellular bacterium that causes canine monocytic ehrlichiosis (CME), a widely distributed infectious disease in dogs with variable clinical severity. While the 16S rRNA gene has been commonly used for detection, limited data are available on the genetic diversity of the immunogenic p28 outer membrane protein gene, particularly in Southeast Asia. Eighty-three canine blood samples were collected from various districts in Ho Chi Minh City, Vietnam, and screened using polymerase chain reaction (PCR) assays targeting the 16S rRNA and p28 genes. Ten representative p28-positive samples were selected for Sanger sequencing. Phylogenetic relationships were inferred using the maximum likelihood method. Genetic variability was assessed through nucleotide and amino acid entropy analysis. Of the 83 samples, 40 (48.19%) were positive for both 16S rRNA and p28 genes. Phylogenetic analysis of p28 sequences revealed three major clades, with all Vietnamese strains clustering within clade 3 and showing a 97%–100% nucleotide identity with global references. Entropy analysis identified 50 high-variability nucleotide sites *H*(*x*): 0.206–1.013 and 20 amino acid positions *H*(*x*): 0.206–1.264, including a prominent substitution at position 145 (S ⟶ D/T), suggesting a potential immunogenic variability. This study provides the first detailed molecular characterization of the *p28* gene in *E. canis* from Vietnam, demonstrating both high genetic conservation and localized variation within clade 3. These findings enhance the understanding of regional strain diversity and may inform future diagnostic or vaccine development efforts targeting the *p28* protein.

## 1. Introduction

*Ehrlichia canis* (*E. canis*) is a small, obligate, intracellular, Gram-negative bacterium that selectively infects mononuclear phagocytes and is the primary causal agent of canine ehrlichiosis, previously referred to as tropical canine pancytopenia [1]. Controlling surface antigenicity may be critical in establishing chronic infections within the host [2]. This pathogen causes clinical symptoms and hematologic irregularities, including fever, lethargy, anorexia, weight loss, cyclic thrombocytopenia, hemorrhage, epistaxis, anemia, and perhaps death [3, 4]. *E. canis* is transmitted by *Rhipicephalus sanguineus* (*R. sanguineus*) and affects the canine mononuclear phagocytic system, namely, monocytes and macrophages [5, 6]. The 16S rRNA gene has been extensively employed in Vietnam to identify *E. canis* in canine populations. This gene functions as a dependable identifier for identifying positive samples owing to its conserved characteristics among *E. canis* strains [7]. Using the 16S rRNA gene in diagnostic tests facilitates the identification of the pathogen in clinically suspected ehrlichiosis patients.

The immunogenic protein genes p28, trp19, and trp36 have been analyzed for the genetic diversity and characteristics of *E. canis* [8–10]. The immunogenic proteins of *E. canis*, such as p28, trp19, trp36, and trp200, have been shown to have significant reactivity with antibodies in the serum of dogs infected with *E. canis* [11]. The 28 kDa outer membrane protein family (p28) is encoded by a multigenic locus, including 25 alleles of the p28 gene of *E. canis* [12]. The recombinant p28 protein is adequate for the serological diagnosis of *E. canis*, and the p28 gene is conserved across all *E. canis* sequences in North America, facilitating the development of vaccine antigens [13]. There is limited information regarding the genetic diversity of isolates in Vietnam, particularly concerning the p28 gene. Therefore, this study aims to investigate the genetic diversity of the p28 gene in canines from Ho Chi Minh City.

## 2. Materials and Methods

### 2.1. Specimen Collection

The Faculty of Veterinary Medicine at Can Tho University approved all experimental procedures involving animals. In addition, consent was obtained to collect biological samples from domestic dogs at Bich Thuy Veterinary Hospital. These procedures adhere to the ethical principles for research involving animals. A team of trained veterinarians carried out all procedures.

Eighty-three blood samples from canine pets or hospital patients in the Ho Chi Minh City region of Vietnam were collected for this study between June and November 2024. Sampling was performed in a nonprobabilistic manner, targeting suspected of infection animals. Three milliliters (mL) of blood were collected by cephalic venipuncture from each animal, placed in tubes containing the anticoagulant ethylene diamine tetra acetic acid (EDTA), stored in a refrigerated container, and transported to the Laboratory of the Faculty of Veterinary Medicine at Can Tho University for further analysis.

### 2.2. Extraction of DNA and PCR Amplification

Genomic DNA was extracted from the dog's whole blood samples using a TopPURE RNA/DNA Viral Extraction Kit (ABT, Vietnam). The DNA sample was eluted in 30 μL EB buffer, and the concentration of purified DNA sample was defined with a NanoDrop 2000 spectrophotometer (Thermo Scientific) at the 260/280 and 260/230 ratios and then aliquoted at a concentration of 100 ng/μL. Finally, the aliquots were stored at – 20^o^C until further use.

Nested PCR for 16S rRNA, the first PCR round amplified a 478 bp segment of *Ehrlichia* 16S rRNA using primers ECC and ECB [14]. The second round amplified a 389 bp fragment using EHCA sense and antisense primers with 1.0 μL of the initial reaction as a template [15]. Amplification of a portion of the gene p28, single PCR using the primers (F: 5′-ATGAATTGCAAAAAAATTCTTATA-3′; R:5′-TTAGAAGTTAAATCTTCCTCC -3′) producing an 843 bp amplicon [9], PCR consisted of a 25 μL reaction mixture with 12 μL GoTaq DNA Polymerase (Promega, USA), 1.0 μL of each 10 μM primer, 8.0 μL deionized water, and 3.0 μL DNA template. The amplification profile consisted of 95°C for 5 min, 30 cycles at 95°C for 30 s, annealing at 55°C for 60 s, and 72°C for 2 min, with a final 5 min at 72°C. The PCR amplicons were stained with 6X GelRed Loading Buffer With Tricolor (ABT, Vietnam), visualized on 1.5% agarose gel electrophoresis under LED light, and photographed. Nuclease-free water was used as a negative control. A 100 bp plus DNA Ladder (Vivantis, Malaysia) was used as a standard for defining the molecular mass of PCR products.

### 2.3. Sequence Alignment and Phylogenetic Entropy Analysis

Ten representative *E. canis*-positive samples were selected for p28 gene sequencing based on DNA quality, PCR band clarity, and diversity in geographic origin, breed, and clinical signs. This selection strategy aimed to capture the genetic variability of circulating strains while minimizing sampling bias. The p28 gene amplicons (843 bp) were purified using the TopPURE PCR/Gel DNA purification kit (ABT, Vietnam) and sequenced by 1^st^ Base DNA Sequencing Division (Selangor, Malaysia). Raw chromatograms were inspected and edited using MEGA v11.0.26. Resulting sequences were subjected to BLAST analysis (NCBI) and subsequently deposited in GenBank.

The phylogenetic tree was constructed using the maximum likelihood (ML) method implemented in MEGA v11.0.26 with 1000 bootstrap replicates and the Kimura-2 parameter model. Sequence alignment was performed with MUSCLE. Trees were annotated using iTOL (https://itol.embl.de). They were analyzing nucleotide similarity ratios using STD v.1.3 software. Nucleotide and amino acid identity were calculated using BioEdit v7.0.5.3, and entropy plots were generated to assess sequence variability.

## 3. Results

### 3.1. Detection and Sample Characteristics

Prior to genetic diversity analysis, all canine blood samples underwent molecular screening for *Ehrlichia canis* using PCR assays targeting both the 16S rRNA and *p28* genes. Among the 83 samples collected from multiple districts across Ho Chi Minh City, 40 (48.19%) were confirmed positive for *E. canis* by amplification of both gene targets. The detailed epidemiological and clinical characteristics of these 40 PCR-confirmed positive cases are summarized in Table 1.

**Figure 1:**
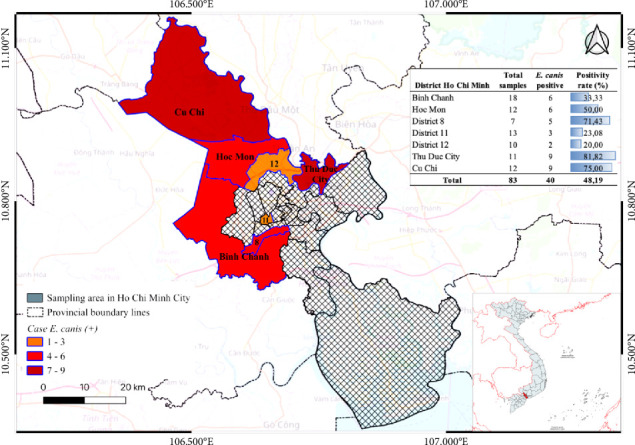
Geographic distribution of *E. canis*-positive cases across districts in Ho Chi Minh City, Vietnam.

Positive cases were geographically distributed across seven administrative districts, including Binh Chanh, Cu Chi, Hoc Mon, Thu Duc City, and Districts 8, 11, and 12. A spatial representation of case density is depicted in Figure 1, with a color gradient illustrating the number of PCR-confirmed infections per district (red: 7–9 cases; orange: 4–6; yellow: 1–3). Notably, the highest positivity rate was recorded in Thu Duc City (81.82%), followed by Cu Chi (75.00%) and District 8 (71.43%).

Infected dogs ranged in age from 1 to 11 years, with a slight male predominance (*n* = 23, 57.5%). Most dogs were either kept indoors or allowed to free-roam, and over half (*n* = 22, 55.0%) belonged to small companion breeds such as Poodles, Chihuahuas, and Shih Tzus. Tick infestation was observed or reported in 22 out of 40 cases (55.0%), while the remaining dogs were free of visible ticks at the time of sampling.

**Figure 2:**
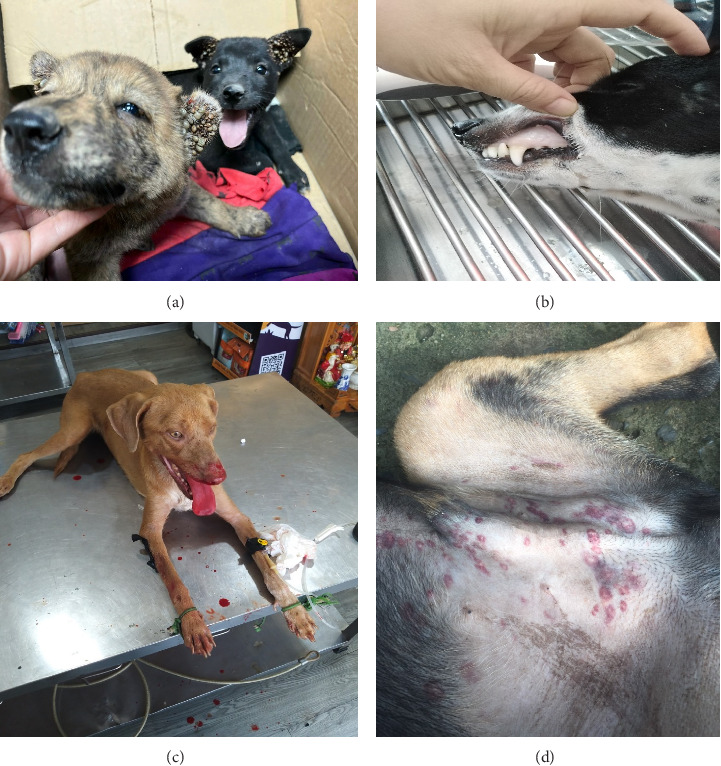
Clinical signs of *E. canis* infection in canine cases. (a) Heavy tick infestation, the primary vector of *E. canis*; (b) pale mucous membranes, suggestive of anemia and potential erythropenia; (c) epistaxis, a clinical manifestation of platelet dysfunction and coagulopathy; (d) petechial and ecchymotic hemorrhages, reflecting vascular endothelial damage and impaired hemostasis.

Clinically, the most frequently reported signs among PCR-confirmed *E. canis*-positive dogs were pale mucous membranes (*n* = 26, 65.0%), generalized body weakness (*n* = 21, 52.5%), bleeding gums (*n* = 18, 45.0%), and subcutaneous hemorrhages (*n* = 17, 42.5%). Other less common manifestations included nosebleeds (*n* = 13, 32.5%), lethargy (*n* = 13, 32.5%), fever (*n* = 12, 30.0%), lymphadenopathy (*n* = 9, 22.5%), and anorexia (*n* = 8, 20.0%). These clinical profiles align with the spectrum of CME, ranging from subclinical infections to severe systemic presentations, potentially influenced by host-related factors such as immune status, nutritional condition, and coinfection with other pathogens. The clinical symptomatology of all PCR-confirmed cases is systematically summarized in Table 1, and Figure 2 illustrates the recorded clinical signs.

Ten representative positive samples (denoted with an asterisk in Table 1) were selected for downstream p28 gene amplification and Sanger sequencing. The resulting sequences (strain IDs: EC/CTU/Vietnam/HCM01–HCM10) were deposited in the GenBank database under accession numbers PQ659473–PQ659482.

### 3.2. Phylogenetic Analysis

**Figure 3:**
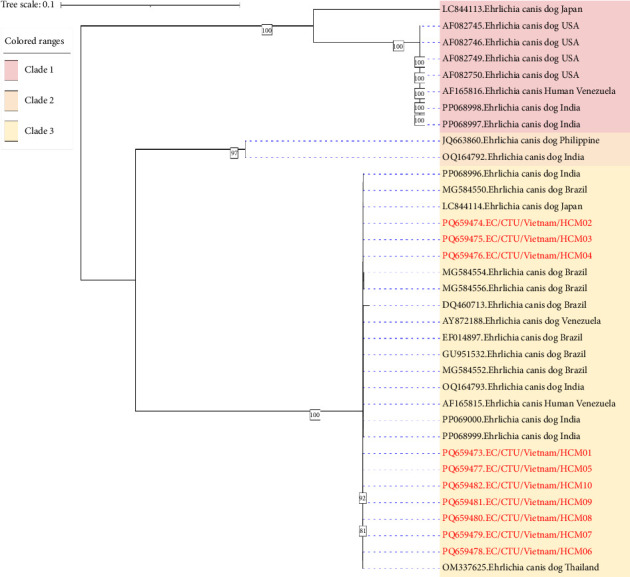
The phylogenetic connections between *E. canis* p28 gene sequences in this work (shown in bold red letters) and those retrieved from GenBank were analyzed using the greatest likelihood approach. The figures on each node represent the bootstrap analysis based on 1000 repetitions. The GenBank accession numbers for the sequences utilized in the phylogenetic trees are also included.

Phylogenetic analysis of p28 gene sequences revealed three major clades supported by high bootstrap values (≥ 80%) (Figure 3). Clade 1 included sequences from the USA (AF082745–AF082750), India (PP068997, PP068998), Venezuela (AF165816), and Japan (LC844113). Clade 2 contained sequences from India (OQ164792) and the Philippines (JQ663860). All 10 Vietnamese sequences in this study clustered into clade 3 and were most similar to EF014897. These clades may indicate evolutionary divergence driven by ecological pressures, vector dynamics, or host immune responses. Their separation suggests potential variation in pathogenicity, vector–host specificity, or regional adaptation. No mixed infections with multiple *E. canis* strains were observed in individual dogs, suggesting dominance of a single circulating strain or limited sensitivity to detect coinfection.

### 3.3. Genetic Analysis

**Figure 4:**
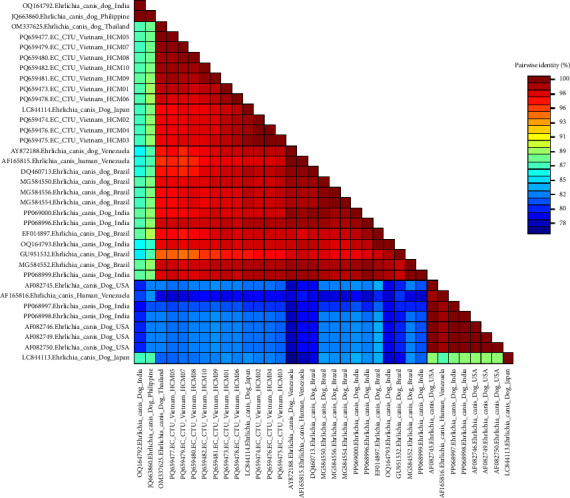
Color-code matrix of pairwise identity scores generated by the alignment of nucleotide sequences of the p28 region of *E. canis* strains isolated in Ho Chi Minh City, Vietnam, and representative isolates from other countries.

The examination of the p28 gene in *E. canis* specimens from Ho Chi Minh City demonstrated significant genetic similarity, ranging from 97% to 100% (red area) (Figure 4), signifying considerable homogeneity. Comparative analysis with samples from South America, Japan, and the USA revealed genetic similarity ranging from 82% to 91% (blue-green area), indicating notable heterogeneity.

### 3.4. Amino Acid Sequence Changes

Samples from Vietnam (accession numbers PQ659473–PQ659482) exhibited amino acid similarity ranging from 95.17% to 98.30% when compared with the reference strain EF014897 (Table 2, Figure S3). These samples showed over 90% similarity to sequences from Brazil (MG584552, MG584553, and MG584556), India (PP068996, PP068999, and PP068900), Thailand (OM337625), and Japan (LC844114). Despite minor amino acid substitutions—such as 48N ⟶ K, 61D ⟶ N, 69T ⟶ S, 71E ⟶ N, 72N ⟶ S, 85S ⟶ N, 125G ⟶ D, 135H ⟶ Y, 136S ⟶ N, 145S ⟶ D/T, 241E ⟶ G, 244S ⟶ T, 246P ⟶ H, and 248N ⟶ D—the p28 gene remained highly conserved. These changes may reflect regional adaptation of E. canis strains and could influence biological behavior or immune recognition.

In contrast, sequences from the USA (AF082745), Japan (LC844113), India (PP068998), and the Philippines (JQ663860) demonstrated lower similarity to EF014897 (74.89%–83.83%), suggesting greater genetic divergence in those regions. This highlights the adaptive plasticity of *E. canis* to different hosts or ecological environments.

While our study focused on genetic variation, the presence of substitutions at predicted immunogenic sites, particularly at high-entropy positions like 145S ⟶ D/T, may have implications for serological assay sensitivity or vaccine design. Further studies are needed to evaluate the impact of these mutations on antigenicity.

### 3.5. Entropy Analysis

**Figure 5:**
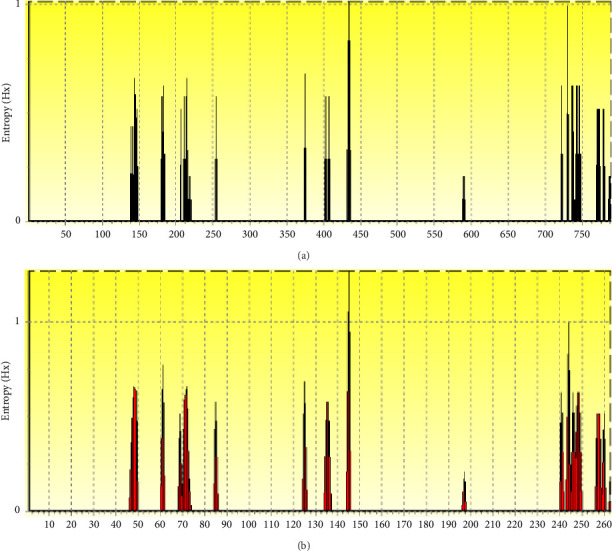
Analysis of entropy *H*(*x*) for *E. canis* p28 sequences utilizing BioEdit software. Entropy plot of nucleotide (a) and amino acid (b) sequence alignments of *E. canis* p28 genes. The peaks denote the significant change at each place within the sequences.

The entropy analysis of the derived nucleotide and amino acid alignments of the p28 sequences identified 51 high-entropy areas in the nucleotide sequence and 24 high-entropy sites in the amino acid sequence (Tables S1-S2). An entropy value over 0.4 signifies a deficiency in conservation at a specific place. Entropy values ranged from 0.206 to 1.01 (nucleotides, Figure 5(a)) and 0.206 to 1.26 (amino acids, Figure 5(b)), with peaks indicating significant genetic variability. A peak of around 1.0 was identified at position 145 for the amino acids derived from p28, indicating areas of substantial genetic variability within this gene. Further analysis of the inferred amino acids of p28 revealed 20 peaks with entropy values exceeding 0.4, concentrated at positions 47–85, 125–147, and 240–260.

## 4. Discussion

This study provides comprehensive insights into the molecular detection, clinical presentation, and genetic diversity of *Ehrlichia canis* among domestic dogs in Ho Chi Minh City, Vietnam. The *E. canis* detection rate of 48.19% is consistent with findings from the Kien Giang province (68.26%) [16], suggesting regional variability likely influenced by ecological conditions, vector distribution, and disease control practices. These findings highlight the clinical complexity of CME, characterized by mucosal pallor, hemorrhagic signs, and systemic weakness—hallmarks of *E. canis*-induced thrombocytopenia and immune-mediated cytopenias. Anorexia and lymphadenopathy were observed in 20%–22.5% of infected dogs CME. Gingival bleeding in 45.0% of dogs underscores the diagnostic value of oral exams in endemic areas. The frequency of fever, lethargy, and lymphadenopathy further supports including *E. canis* in differential diagnoses of febrile illnesses with hematological changes. These patterns align with global clinical reports [3, 4], suggesting comparable pathogenicity of Vietnamese strains.

Tick infestation was observed in 55% of positive cases, supporting the recognized role of *R. sanguineus* as the primary vector. Although systematic acarological identification was not performed, field observations confirmed the frequent presence of *R. sanguineus* on infected dogs. This aligns with prior reports of *R. sanguineus* distribution in Vietnam, where a nationwide prevalence of 1.8% was documented [7]. These findings highlight the ecological complexity of vector–host interactions and underscore the potential for both endemic and episodic transmissions within urban and periurban environments.

The p28 gene encodes a 28 kDa surface protein that promotes bacterial adherence to host cell membranes and aids immune evasion. This protein serves as a vital antigen, provoking an immunological response, with antibodies often emerging around 2 weeks after infection [11, 17]. Our findings reveal that clade 3 is the major group found in many nations based on p28 gene sequences, exhibiting considerable genetic conservation in the structure of *E. canis*. These results align with [18], emphasizing that clade 3, which includes genomes from Vietnam and other nations, has significant genetic similarities, demonstrating the worldwide dispersal of this clade. This distribution may be affected by the worldwide pet trade, human migration, and climatic conditions that enhance vector viability.

The differentiation among clades 1, 2, and 3 suggests the presence of multiple *E. canis* lineages shaped by ecological factors, vector distributions, and host-related pressures in distinct geographic regions. These phylogenetic groupings may also be associated with differences in virulence, immune evasion strategies, or vector competence, although such functional correlations were not assessed in this study. The exclusive clustering of Vietnamese strains within clade 3 may reflect local adaptation to regional transmission dynamics. Clade 1, including sequences from the USA, Japan, Venezuela, and areas of India, presumably signifies a lineage suited to certain climatic circumstances [19, 20]. Clade 2, despite its restricted size, has sequences from the Philippines and India, suggesting a regionally developed lineage shaped by selection forces [21–23]. Previous studies proposed that fluctuations in immune-related proteins may result from increased selection pressures, which in turn influence bacterial adaptation mechanisms and immune evasion strategies. The p28 gene functions as a crucial diagnostic marker for *E. canis*; nevertheless, the scarcity of p28 sequences in GenBank impedes a comprehensive investigation of genetic diversity. This research investigates the genetic diversity and conservation of *E. canis* in Ho Chi Minh City, Vietnam, pinpointing local strains within clade 3, the most widely dispersed clade worldwide.

The genetic study demonstrated significant homogeneity (97%–100%) across local strains, corroborating the findings of [24], who observed the same patterns in areas with stable tick populations. This uniformity may be elucidated by geographic factors, vector species, and varying epidemiological characteristics between locations. Research conducted by [5, 25] in South America revealed genetic clustering in subtropical and tropical regions associated with tick movement patterns. The humid tropical climate of Vietnam, marked by persistently elevated temperatures, facilitates the multiplication of *Rhipicephalus sanguineus*, increasing the danger of *E. canis* transmission.

The p28 gene is essential for host immunological interactions, allowing *E. canis* to acclimatize to various environmental circumstances. Prior studies, such as those by [13], indicated that the diversity of the p28 gene affects pathogen infectivity and pathogenicity. The genetic uniformity of *E. canis* in Vietnam indicates ongoing endemicity with the restricted influx of divergent strains. This discovery corresponds with regional research, including [4] in Thailand and [26] in Malaysia, reporting significant genetic homogeneity in *E. canis* populations. The authors of [25] noted considerable genetic similarities among *E. canis* strains in Brazil, comparable to those in Vietnam in our research.

The amino acid sequence analysis revealed alterations that may influence pathogen adaptability and pathogenicity. The genetic variability of the p28 gene enables immune evasion and affects the differentiation of worldwide *E. canis* populations. These results align with the observations of [9, 27, 28] who documented analogous modifications in the amino acid sequences of the p28 gene. Although the p28 gene is conserved primarily, significant amino acid changes underscore *E. canis*'s resilience across many geographical locations and ecological niches.

Entropy analysis revealed significant heterogeneity at amino acid positions 47–85, 125–147, and 240–260. These findings align with [9, 28], who noted polymorphism-associated variations in amino acid sequences. The authors of [18] documented entropy values between 0.15 and 1.00 in investigations of p28 gene polymorphism. The authors of [9] proposed that these divergences may arise from selection pressures exerted on infections and hosts. High-entropy zones indicate active evolutionary pressures, presumably influenced by vector adaptation and host–pathogen dynamics.

This work offers significant insights into the genetic architecture and evolutionary processes of *E. canis* in Vietnam. The genetic variety and adaptability discovered underscore the need for ongoing surveillance to comprehend pathogen dynamics and guide appropriate control tactics.

## 5. Conclusion

This study is the first publication illustrating the genetic variety of *E. canis* based on the p28 gene sequence in Ho Chi Minh City, Vietnam. Our data demonstrate that the genetic diversity of the *E. canis* p28 gene is predominantly not preserved, both in Vietnam and worldwide. These findings may improve the comprehension of molecular phylogeny and diversity among *E. canis* p28 genes extracted in Vietnam. The processes behind these genetic divergences in *E. canis* are still unidentified. Nonetheless, as these variations arise in genes that encode proteins, the immunological pressure from vertebrate hosts and adaptability to invertebrate hosts may significantly influence the survival of these bacteria.

## Figures and Tables

**Table 1:** Epidemiological and clinical characteristics of *E. canis*-positive dog samples from Ho Chi Minh City, Vietnam.

Sample ID	Age (year)	Sex	District	Raising method	Tick infestation	Breed	Clinical symptoms (coded)
HCM01^∗^	2	Male	Binh Chanh	Outdoor	Yes	Poodle	1; 2; 3; 4; 5
HCM02^∗^	5	Female	Hoc Mon	Free-roaming	Yes	Shih Tzu	3; 4
HCM03^∗^	3	Male	District 8	Free-roaming	Yes	Poodle	1; 2
HCM04^∗^	2	Female	District 11	Indoor	No	Mixed	3; 4; 5
HCM05^∗^	3	Female	District 12	Free-roaming	No	Shih Tzu	1; 2; 4
HCM06^∗^	5	Male	Thu Duc City	Indoor	Yes	Labrador	1; 2; 4; 3; 5
HCM07^∗^	4	Female	Thu Duc City	Indoor	Yes	Chihuahua	1; 2; 4; 5
HCM08^∗^	1	Female	Hoc Mon	Free-roaming	No	Phu Quoc	1; 2
HCM09^∗^	3	Female	Cu Chi	Indoor	No	Chihuahua	3; 5; 4
HCM10^∗^	1	Male	Thu Duc City	Indoor	Yes	Beagle	1; 2; 3; 5
HCM11	8	Female	District 8	Outdoor	Yes	Chihuahua	2
HCM12	5	Male	Cu Chi	Outdoor	No	Mixed	2; 6; 7
HCM13	1	Female	District 12	Free-roaming	No	Poodle	2; 6; 7
HCM14	4	Female	Cu Chi	Free-roaming	No	Beagle	3; 5
HCM15	7	Male	Thu Duc City	Indoor	No	Chihuahua	4; 8
HCM16	7	Male	Cu Chi	Free-roaming	No	Mixed	2; 6; 7
HCM17	8	Female	Cu Chi	Outdoor	Yes	Labrador	1; 9
HCM18	7	Male	Hoc Mon	Indoor	No	Phu Quoc	3; 5
HCM19	2	Male	District 11	Indoor	No	Labrador	4; 8
HCM20	10	Male	Thu Duc City	Indoor	No	Mixed	2; 6; 7
HCM21	6	Male	District 11	Indoor	No	Phu Quoc	2; 6; 8
HCM22	8	Female	Binh Chanh	Indoor	No	Chihuahua	2; 6; 9
HCM23	7	Male	Binh Chanh	Outdoor	No	Poodle	2; 6; 7
HCM24	10	Male	Thu Duc City	Indoor	Yes	Shih Tzu	1; 9
HCM25	10	Female	Thu Duc City	Indoor	Yes	Poodle	1; 9
HCM26	10	Female	Hoc Mon	Indoor	No	Beagle	4; 8
HCM27	7	Male	Cu Chi	Outdoor	No	Chihuahua	3; 5
HCM28	11	Female	Cu Chi	Free-roaming	No	Beagle	4; 8
HCM29	11	Male	Hoc Mon	Indoor	Yes	Beagle	1; 9
HCM30	8	Male	Thu Duc City	Free-roaming	No	Labrador	2; 6; 7
HCM31	4	Female	Binh Chanh	Outdoor	No	Labrador	4; 8
HCM32	1	Female	Cu Chi	Indoor	No	Chihuahua	4; 7
HCM33	5	Male	District 8	Outdoor	Yes	Labrador	2
HCM34	10	Male	District 8	Outdoor	No	Chihuahua	2; 6; 7
HCM35	6	Male	Hoc Mon	Free-roaming	No	Shih Tzu	2; 6; 7
HCM36	9	Female	Binh Chanh	Free-roaming	No	Chihuahua	4; 7
HCM37	8	Male	Binh Chanh	Free-roaming	Yes	Phu Quoc	2; 6; 7
HCM38	6	Female	District 8	Indoor	No	Poodle	3; 5
HCM39	6	Female	Cu Chi	Free-roaming	Yes	Phu Quoc	1; 9
HCM40	2	Female	Thu Duc City	Outdoor	Yes	Shih Tzu	1; 7

*Note:* Samples marked with an asterisk (^∗^) were selected for p28 gene sequencing (accession numbers PQ659473–PQ659482). Clinical symptoms were numerically coded as follows: 1 = bleeding gums; 2 = pale mucous membranes; 3 = subcutaneous hemorrhages; 4 = body weakness; 5 = nosebleeds; 6 = fever; 7 = lethargy; 8 = lymphadenopathy; 9 = anorexia. All 40 samples tested positive for both 16S rRNA and p28 genes.

**Table 2:** Amino acid differences in the partial sequence of the polypeptide deduced from p28 among *E*. *canis* sequences.

No.	Strains *E. canis*	Identity percentage (%)	Amino acid positions																					
			21	23	47	48	49	61	69	71	72	73	85	125	135	136	145	197	241	243	244	246	247	248
1	EF014897. *E. canis* Brazil	100.0	T	-	S	N	P	D	T	E	N	I	S	G	H	S	S	P	E	-	S	P	K	N
2	PP068999. *E. canis* India	99.57	.	-	.	.	.	.	.	.	.	.	.	.	.	.	.	.	G	-	.	.	.	.
3	PP068996. *E. canis* India	98.72	.	-	.	.	.	.	.	.	.	.	.	.	.	.	N	.	G	-	N	.	.	.
4	MG584552. *E. canis* Brazil	99.57	.	-	.	.	.	.	.	.	.	.	.	.	.	.	.	.	G	-	.	.	.	.
5	PP069000. *E. canis* India	96.60	.	-	R	.	S	N	S	.	.	.	N	.	.	.	.	.	G	-	N	.	E	.
6	MG584553. *E. canis* Brazil	97.45	.	-	R	.	S	N	S	.	.	.	N	.	.	.	N	.	.	-	.	.	.	.
7	MG584556. *E. canis* Brazil	96.17	.	-	R	.	S	N	S	.	.	T	N	.	.	.	D	S	G	-	N	.	.	.
8	LC844114. *E. canis* Japan	97.02	.	-	.	.	T	G	S	N	S	.	.	.	.	N	D	.	G	-	.	.	.	.
9	OM337625. *E. canis* Thailand	95.74	.	-	.	K	.	.	S	.	S	.	.	D	Y	.	T	.	G	-	T	H	.	D
10	PQ659473.EC/CTU/Vietnam/HCM01	97.87	.	-	.	K	.	.	S	.	S	.	.	D	Y	.	T	.	.	-	.	.	.	.
11	PQ659474.EC/CTU/Vietnam/HCM02	98.30	.	-	.	.	.	.	S	N	S	.	.	.	.	N	D	.	.	-	.	.	.	.
12	PQ659475.EC/CTU/Vietnam/HCM03	97.87	.	-	.	.	.	.	S	N	S	.	.	.	.	N	D	.	G	-	.	.	.	.
13	PQ659476.EC/CTU/Vietnam/HCM04	98.30	.	-	.	.	.	.	S	N	S	.	.	.	.	N	D	.	.	-	.	.	.	.
14	PQ659477.EC/CTU/Vietnam/HCM05	96.17	.	-	.	K	.	.	S	.	S	.	.	D	Y	.	.	.	G	-	T	H	.	D
15	PQ659478.EC/CTU/Vietnam/HCM06	97.02	.	-	.	K	.	.	S	N	S	.	N	D	.	N	T	.	.	-	.	.	.	.
16	PQ659479.EC/CTU/Vietnam/HCM07	95.74	.	-	.	K	.	.	S	.	S	.	.	D	Y	.	D	.	G	-	T	H	.	D
17	PQ659480.EC/CTU/Vietnam/HCM08	95.32	.	-	.	K	.	N	S	.	S	.	.	D	.	.	D	.	G	-	T	H	.	D
18	PQ659481.EC/CTU/Vietnam/HCM09	96.17	.	-	.	.	.	.	S	.	S	.	N	D	.	.	.	.	G	-	T	H	.	D
19	PQ659482.EC/CTU/Vietnam/HCM10	95.74	.	-	.	K	.	N	S	.	S	.	.	D	Y	.	.	.	G	-	T	H	.	D
20	JQ663860. *E. canis* Philippine	83.83	.	-	R	.	.	N	.	G	S	.	I	D	.	N	.	L	.	-	.	Q	.	.
21	LC844113. *E. canis* Japan	77.45	.	-	R	.	S	N	-	C	S	E	N	D	G	-	Q	.	D	-	A	.	.	.
22	PP068998. *E. canis* India	74.89	M	G	.	K	S	.	-	H	A	D	N	D	.	-	A	.	N	T	T	S	G	P
23	AF082745. *E. canis* USA	74.89	M	G	.	K	S	.	-	H	A	D	N	D	.	-	A	.	N	T	T	S	G	P

*Note:* Position based on the sequence of (EF014897); the dots (.), conserved region; the (-) represents an ‘‘indel” base.

## Data Availability

The data that support the findings of this study are available in the supplementary material of this article.

## Supporting Information

Figure S1: 16S rRNA gene amplification by nPCR. The 389 bp nPCR amplification product of 16S rRNA gene as visualized in 1.5% agarose gel containing GelRed loading buffer.

Figure S2: The p28 gene amplification by PCR. The 843 bp PCR amplification product of p28 gene as visualized in 1.5% agarose gel containing GelRed loading buffer.

Figure S3: Amino acid differences in the partial sequence of the polypeptide deduced from p28 among *Ehrlichia canis* sequences. Position based on the sequence of Brazil (EF014897); the dots (.) represent conserved region; the (-) represents an ‘‘indel” base.

Table S1: *E. canis* gene p28 nucleotide entropy (*H* (x)). The *E. canis* strains selected for entropy comparison include samples from various countries, reflecting the geographical diversity of this species (Brazil: EF014897, MG584552, MG584553, MG584556; India: PP068999, PP068996, PP069000; Japan: LC844114; Thailand: OM337625; Vietnam: PQ659473–PQ659482). Position based on the sequence of Brazil (EF014897).

Table S2: *E. canis* gene p28 acid amine entropy (*H* (x)). The *E. canis* strains selected for entropy comparison include samples from various countries, reflecting the geographical diversity of this species (Brazil: EF014897, MG584552, MG584553, MG584556; India: PP068999, PP068996, PP069000; Japan: LC844114; Thailand: OM337625; Vietnam: PQ659473–PQ659482). Position based on the sequence of Brazil (EF014897).

## References

[1] Immelman A., Button C. (1973). Ehrlichia Canis Infection (Tropical Canine Pancytopaenia or Canine Rickettsiosis). *Journal of the South African Veterinary Association*.

[2] Parola P., Cornet J. P., Sanogo Y. O. (2003). Detection of Ehrlichia spp., Anaplasma spp., Rickettsia spp., and Other Eubacteria in Ticks From the Thai-Myanmar Border and Vietnam. *Journal of Clinical Microbiology*.

[3] Saito T. B., Walker D. H. (2016). Ehrlichioses: An Important One Health Opportunity. *Veterinary Sciences*.

[4] Nambooppha B., Rittipornlertrak A., Tattiyapong M. (2018). Two Different Genogroups of Ehrlichia Canis From Dogs in Thailand Using Immunodominant Protein Genes. *Infection, Genetics and Evolution*.

[5] Aguiar D. M., Cavalcante G. T., Pinter A., Gennari S. M., Camargo L. M. A., Labruna M. B. (2007). Prevalence of Ehrlichia Canis (Rickettsiales: Anaplasmataceae) in Dogs and *Rhipicephalus sanguineus* (Acari: Ixodidae) Ticks From Brazil. *Journal of Medical Entomology*.

[6] Dantas-Torres F., Latrofa M. S., Ramos R. A. N. (2018). Biological Compatibility Between Two Temperate Lineages of Brown Dog Ticks, *Rhipicephalus sanguineus* (Sensu Lato). *Parasites & Vectors*.

[7] Nguyen V. L., Colella V., Iatta R., Bui K. L., Dantas-Torres F., Otranto D. (2019). Ticks and Associated Pathogens From Dogs in Northern Vietnam. *Parasitology Research*.

[8] Hsieh Y. C., Lee C. C., Tsang C. L., Chung Y. T. (2010). Detection and Characterization of Four Novel Genotypes of *Ehrlichia canis* From Dogs. *Veterinary Microbiology*.

[9] Nakaghi A. C. H., Machado R. Z., Ferro J. A. (2010). Sensitivity Evaluation of a Single-Step PCR Assay Using Ehrlichia Canis p28 Gene as a Target and Its Application in Diagnosis of *Canine ehrlichiosis*. *Revista Brasileira de Parasitologia Veterinaria*.

[10] Cicuttin G. L., De Salvo M. N., Gury Dohmen F. E. (2016). Molecular Characterization of *Ehrlichia canis* Infecting Dogs, Buenos Aires. *Ticks and Tick-Borne Diseases*.

[11] McBride J. W., Corstvet R. E., Gaunt S. D., Boudreaux C., Guedry T., Walker D. H. (2003). Kinetics of Antibody Response to *Ehrlichia canis* Immunoreactive Proteins. *Infection and Immunity*.

[12] Long S. W., Zhang X. F., Qi H., Standaert S., Walker D. H., Yu X. J. (2002). Antigenic Variation of *Ehrlichia chaffeensis* Resulting From Differential Expression of the 28-Kilodalton Protein Gene Family. *Infection and Immunity*.

[13] McBride J. W., Yu X. J., Walker D. H. (2000). A Conserved, Transcriptionally Active p28 Multigene Locus of *Ehrlichia canis*. *Gene*.

[14] Dawson J. E., Stallknecht D. E., Howerth E. W. (1994). Susceptibility of White-Tailed Deer (*Odocoileus virginianus*) to Infection With *Ehrlichia chaffeensis*, the Etiologic Agent of Human Ehrlichiosis. *Journal of Clinical Microbiology*.

[15] Wen B., Rikihisa Y., Mott J. M. (1997). Comparison of Nested PCR With Immunofluorescent-Antibody Assay for Detection of *Ehrlichia canis* Infection in Dogs Treated With Doxycycline. *Journal of Clinical Microbiology*.

[16] Lê T. Đ., Nguyễn Đ. C., Dương A. T., Nguyễn K. T., Lý T. L. K. (2021). Đặc Điểm Và Phương Pháp Chẩn Đoán Bệnh Do Ehrlichia Canis Gây Ra Trên Chó Tại Thành Phố Rạch Giá, Tỉnh Kiên Giang. *Can Tho University Journal of Science*.

[17] Wagner E. R., Bremer W. G., Rikihisa Y. (2004). Development of a p28-Based PCR Assay for *Ehrlichia chaffeensis*. *Molecular and Cellular Probes*.

[18] Costa R. L., Paulino P. G., da Silva C. B. (2019). Molecular Characterization of *Ehrlichia canis* From Naturally Infected Dogs From the State of Rio De Janeiro. *Brazilian Journal of Microbiology*.

[19] Ohashi N., Rikihisa Y., Unver A. (2001). Analysis of Transcriptionally Active Gene Clusters of Major Outer Membrane Protein Multigene Family Ine*hrlichia Canisande. Chaffeensis*. *Infection and Immunity*.

[20] Beall M. J., Alleman A. R., Breitschwerdt E. B. (2012). Seroprevalence of Ehrlichia Canis, *Ehrlichia chaffeensis* and *Ehrlichia ewingii* in Dogs in North America. *Parasites & Vectors*.

[21] Ybañez A. P., Ybañez R. H. D., Villavelez R. R. (2016). Retrospective Analyses of Dogs Found Serologically Positive for *Ehrlichia canis* in Cebu, Philippines From 2003 to 2014. *Veterinary World*.

[22] Maekawa N., Konnai S., Balbin M. M. (2018). Molecular Detection and Phylogenetic Analysis of *Ehrlichia canis* in a Philippine Dog. *Ticks and Tick-Borne Diseases*.

[23] Aguiar D. M., Zhang X., Melo A. L. T. (2013). Genetic Diversity of Ehrlichia Canis in Brazil. *Veterinary Microbiology*.

[24] Inokuma H., Parola P., Raoult D., Brouqui P. (2001). Molecular Survey of Ehrlichia Infection in Ticks From Animals in Yamaguchi Prefecture, Japan. *Veterinary Parasitology*.

[25] Labruna M. B., McBride J. W., Camargo L. M. A. (2007). A Preliminary Investigation of Ehrlichia Species in Ticks, Humans, Dogs, and Capybaras From Brazil. *Veterinary Parasitology*.

[26] Nazari M., Lim S. Y., Watanabe M., Sharma R. S., Cheng N. A., Watanabe M. (2013). Molecular Detection of Ehrlichia Canis in Dogs in Malaysia. *PLoS Neglected Tropical Diseases*.

[27] Alves R. N., Rieck S. E., Ueira-Vieira C., Labruna M. B., Beletti M. E. (2014). Isolation, in Vitro Propagation, Genetic Analysis, and Immunogenic Characterization of an Ehrlichia Canis Strain From Southeastern Brazil. *Journal of Veterinary Science*.

[28] Poolsawat N., Nooroong P., Junsiri W. (2023). Ehrlichia Canis: Molecular Characterization and Genetic Diversity Based on the p28 and trp36 Genes. *Research in Veterinary Science*.

