# Supplementary material for: Molecular Investigation of the p28 Gene of Ehrlichia canis in Infected Dogs From Ho Chi Minh City, Vietnam
Source: Vet Med Int. 2025 Oct 6;2025:8884821. doi: 10.1155/vmi/8884821 (PMC12517983; doi:10.1155/vmi/8884821)
Supplement: Supporting Information — Additional supporting information can be found online in the Supporting Information section. [file 8884821.f1.docx]

**Supplementary**

**Supplementary figures:**


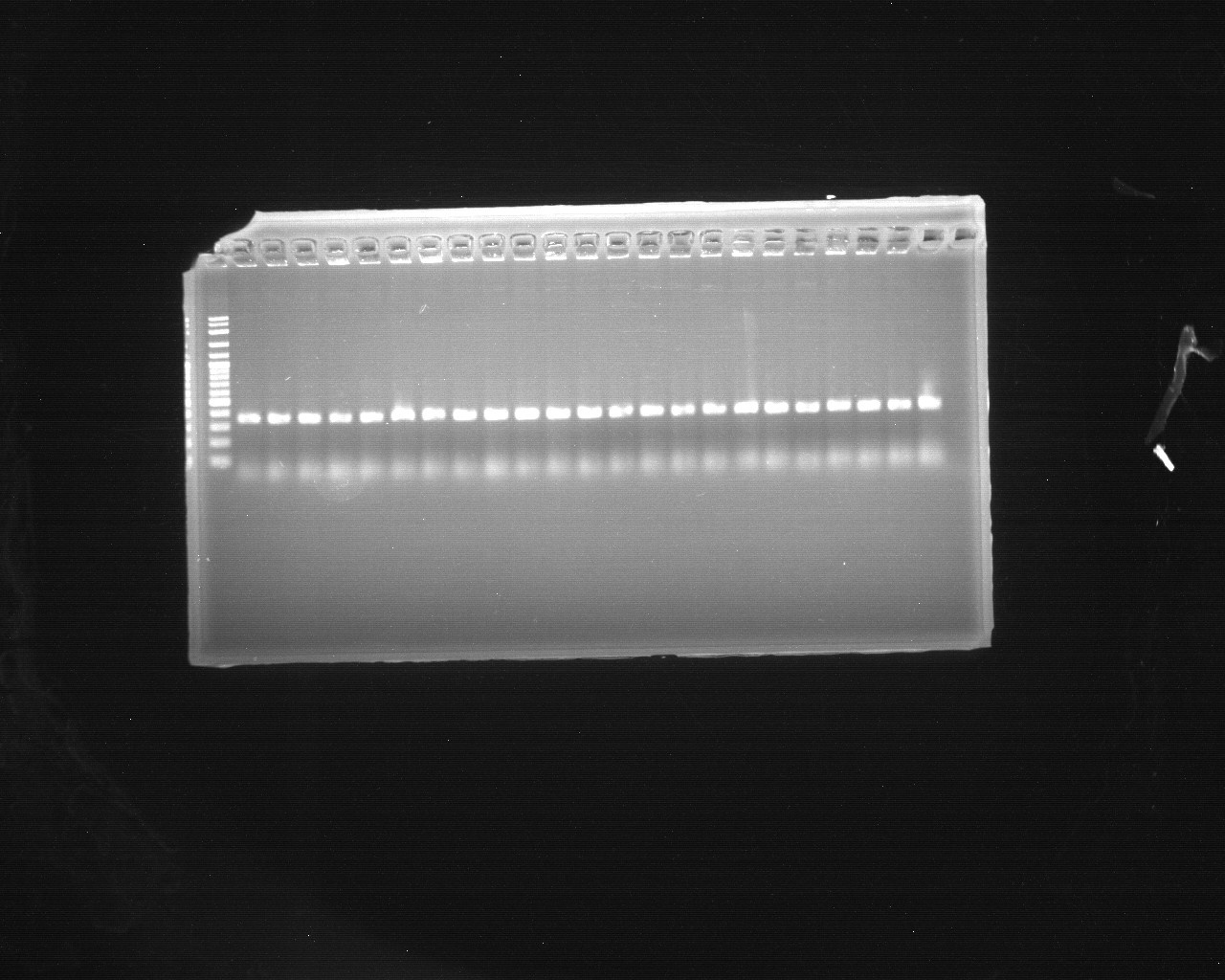


**Figure S1:** 16S rRNA gene amplification by PCR. The 389bp nPCR amplification product of 16S rRNA gene as visualized in 1.5% agarose gel containing GelRed loading buffer.


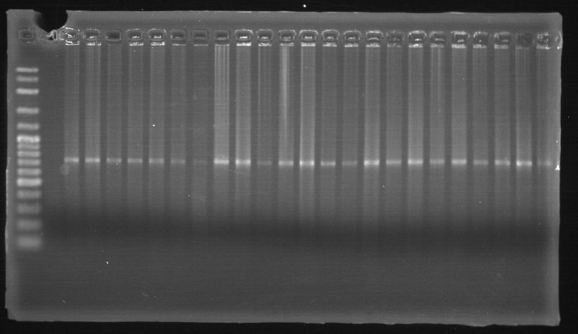


**Figure S2:** The p28 gene amplification by PCR. The 843bp PCR amplification product of p28 gene as visualized in 1.5% agarose gel containing GelRed loading buffer.


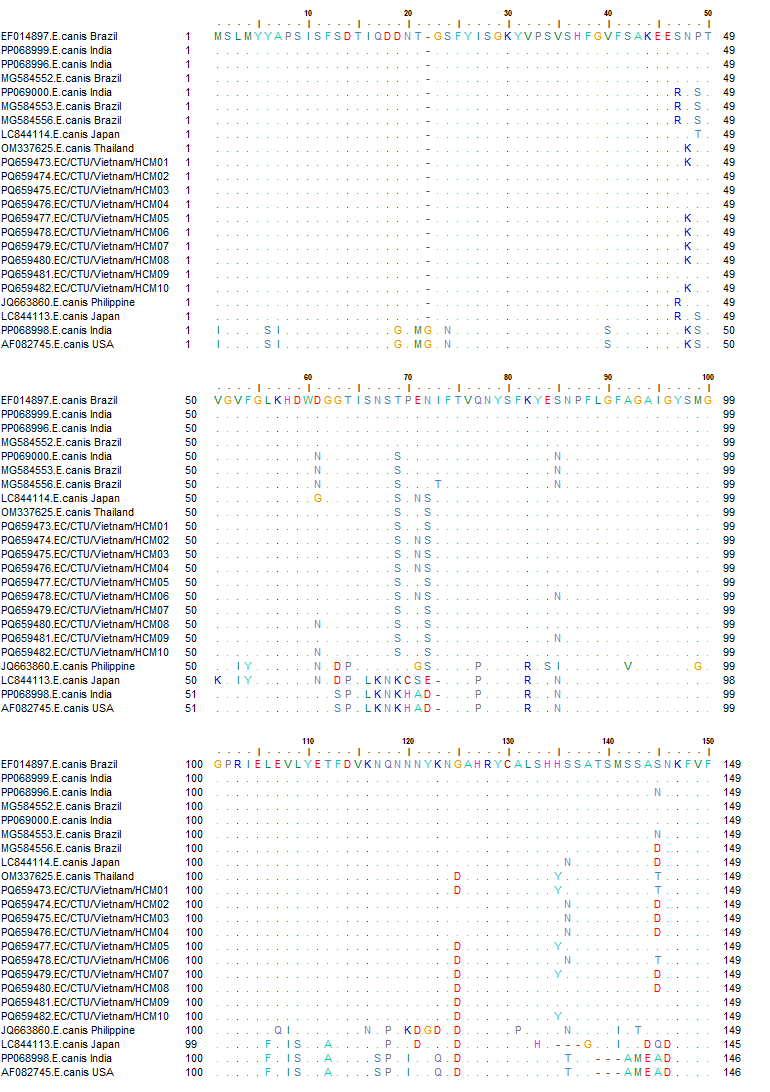


**Figure S3:** Amino acid differences in the partial sequence of the polypeptide deduced from p28 among *Ehrlichia canis* sequences. Position based on the sequence of Brazil (EF014897); The dots (.), conserved region; The (-) represents an ‘‘indel” base


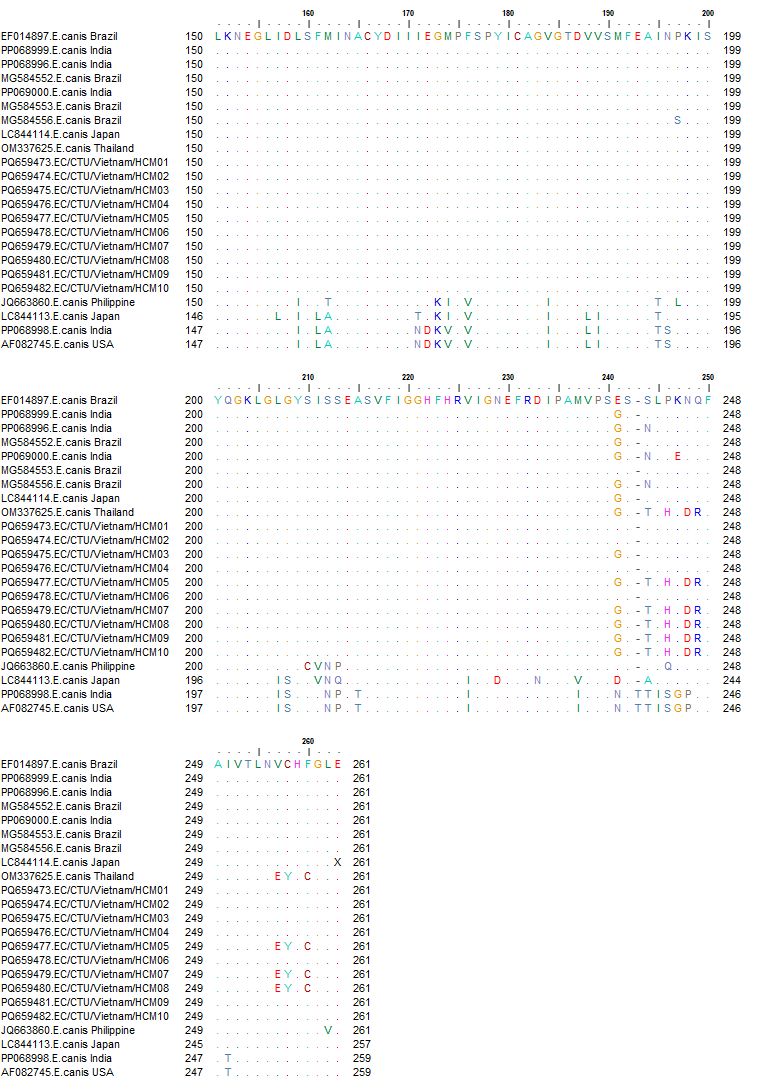
**Figure S3:** Amino acid differences in the partial sequence of the polypeptide deduced from p28 among *Ehrlichia canis* sequences. Position based on the sequence of Brazil (EF014897); The dots (.), conserved region; The (-) represents an ‘‘indel” base (Continued)

**Supplementary Table:**

**Table S1**: *E. canis* gene p28 nucleotide Entropy (*H* (x)). The *E. canis* strains selected for entropy comparison include samples from various countries, reflecting the geographical diversity of this species (Brazil: EF014897, MG584552, MG584553, MG584556; India: PP068999, PP068996, PP069000; Japan: LC844114; Thailand: OM337625; Vietnam: PQ659473 - PQ659482). Position based on the sequence of Brazil (EF014897)

| Position nucleotide | Entropy (*H* (x)) |
| --- | --- |
| Position 1 | 0.000 |
| Position 2 | 0.000 |
| Position 3 | 0.000 |
| Position 4 | 0.000 |
| Position 5 | 0.000 |
| Position 6 | 0.000 |
| Position 7 | 0.000 |
| Position 8 | 0.000 |
| Position 9 | 0.000 |
| Position 10 | 0.000 |
| Position 11 | 0.000 |
| Position 12 | 0.000 |
| Position 13 | 0.000 |
| Position 14 | 0.000 |
| Position 15 | 0.000 |
| Position 16 | 0.000 |
| Position 17 | 0.000 |
| Position 18 | 0.000 |
| Position 19 | 0.000 |
| Position 20 | 0.000 |
| Position 21 | 0.000 |
| Position 22 | 0.000 |
| Position 23 | 0.000 |
| Position 24 | 0.000 |
| Position 25 | 0.000 |
| Position 26 | 0.000 |
| Position 27 | 0.000 |
| Position 28 | 0.000 |
| Position 29 | 0.000 |
| Position 30 | 0.000 |
| Position 31 | 0.000 |
| Position 32 | 0.000 |
| Position 33 | 0.000 |
| Position 34 | 0.000 |
| Position 35 | 0.000 |
| Position 36 | 0.000 |
| Position 37 | 0.000 |
| Position 38 | 0.000 |
| Position 39 | 0.000 |
| Position 40 | 0.000 |
| Position 41 | 0.000 |
| Position 42 | 0.000 |
| Position 43 | 0.000 |
| Position 44 | 0.000 |
| Position 45 | 0.000 |
| Position 46 | 0.000 |
| Position 47 | 0.000 |
| Position 48 | 0.000 |
| Position 49 | 0.000 |
| Position 50 | 0.000 |
| Position 51 | 0.000 |
| Position 52 | 0.000 |
| Position 53 | 0.000 |
| Position 54 | 0.000 |
| Position 55 | 0.000 |
| Position 56 | 0.000 |
| Position 57 | 0.000 |
| Position 58 | 0.000 |
| Position 59 | 0.000 |
| Position 60 | 0.000 |
| Position 61 | 0.000 |
| Position 62 | 0.000 |
| Position 63 | 0.000 |
| Position 64 | 0.000 |
| Position 65 | 0.000 |
| Position 66 | 0.000 |
| Position 67 | 0.000 |
| Position 68 | 0.000 |
| Position 69 | 0.000 |
| Position 70 | 0.000 |
| Position 71 | 0.000 |
| Position 72 | 0.000 |
| Position 73 | 0.000 |
| Position 74 | 0.000 |
| Position 75 | 0.000 |
| Position 76 | 0.000 |
| Position 77 | 0.000 |
| Position 78 | 0.000 |
| Position 79 | 0.000 |
| Position 80 | 0.000 |
| Position 81 | 0.000 |
| Position 82 | 0.000 |
| Position 83 | 0.000 |
| Position 84 | 0.000 |
| Position 85 | 0.000 |
| Position 86 | 0.000 |
| Position 87 | 0.000 |
| Position 88 | 0.000 |
| Position 89 | 0.000 |
| Position 90 | 0.000 |
| Position 91 | 0.000 |
| Position 92 | 0.000 |
| Position 93 | 0.000 |
| Position 94 | 0.000 |
| Position 95 | 0.000 |
| Position 96 | 0.000 |
| Position 97 | 0.000 |
| Position 98 | 0.000 |
| Position 99 | 0.000 |
| Position 100 | 0.000 |
| Position 101 | 0.000 |
| Position 102 | 0.000 |
| Position 103 | 0.000 |
| Position 104 | 0.000 |
| Position 105 | 0.000 |
| Position 106 | 0.000 |
| Position 107 | 0.000 |
| Position 108 | 0.000 |
| Position 109 | 0.000 |
| Position 110 | 0.000 |
| Position 111 | 0.000 |
| Position 112 | 0.000 |
| Position 113 | 0.000 |
| Position 114 | 0.000 |
| Position 115 | 0.000 |
| Position 116 | 0.000 |
| Position 117 | 0.000 |
| Position 118 | 0.000 |
| Position 119 | 0.000 |
| Position 120 | 0.000 |
| Position 121 | 0.000 |
| Position 122 | 0.000 |
| Position 123 | 0.000 |
| Position 124 | 0.000 |
| Position 125 | 0.000 |
| Position 126 | 0.000 |
| Position 127 | 0.000 |
| Position 128 | 0.000 |
| Position 129 | 0.000 |
| Position 130 | 0.000 |
| Position 131 | 0.000 |
| Position 132 | 0.000 |
| Position 133 | 0.000 |
| Position 134 | 0.000 |
| Position 135 | 0.000 |
| Position 136 | 0.436 |
| Position 137 | 0.000 |
| Position 138 | 0.436 |
| Position 139 | 0.000 |
| Position 140 | 0.000 |
| Position 141 | 0.658 |
| Position 142 | 0.515 |
| Position 143 | 0.436 |
| Position 144 | 0.515 |
| Position 145 | 0.000 |
| Position 146 | 0.000 |
| Position 147 | 0.000 |
| Position 148 | 0.000 |
| Position 149 | 0.000 |
| Position 150 | 0.000 |
| Position 151 | 0.000 |
| Position 152 | 0.000 |
| Position 153 | 0.000 |
| Position 154 | 0.000 |
| Position 155 | 0.000 |
| Position 156 | 0.000 |
| Position 157 | 0.000 |
| Position 158 | 0.000 |
| Position 159 | 0.000 |
| Position 160 | 0.000 |
| Position 161 | 0.000 |
| Position 162 | 0.000 |
| Position 163 | 0.000 |
| Position 164 | 0.000 |
| Position 165 | 0.000 |
| Position 166 | 0.000 |
| Position 167 | 0.000 |
| Position 168 | 0.000 |
| Position 169 | 0.000 |
| Position 170 | 0.000 |
| Position 171 | 0.000 |
| Position 172 | 0.000 |
| Position 173 | 0.000 |
| Position 174 | 0.000 |
| Position 175 | 0.000 |
| Position 176 | 0.000 |
| Position 177 | 0.000 |
| Position 178 | 0.576 |
| Position 179 | 0.206 |
| Position 180 | 0.624 |
| Position 181 | 0.000 |
| Position 182 | 0.000 |
| Position 183 | 0.000 |
| Position 184 | 0.000 |
| Position 185 | 0.000 |
| Position 186 | 0.000 |
| Position 187 | 0.000 |
| Position 188 | 0.000 |
| Position 189 | 0.000 |
| Position 190 | 0.000 |
| Position 191 | 0.000 |
| Position 192 | 0.000 |
| Position 193 | 0.000 |
| Position 194 | 0.000 |
| Position 195 | 0.000 |
| Position 196 | 0.000 |
| Position 197 | 0.000 |
| Position 198 | 0.000 |
| Position 199 | 0.000 |
| Position 200 | 0.000 |
| Position 201 | 0.000 |
| Position 202 | 0.000 |
| Position 203 | 0.515 |
| Position 204 | 0.000 |
| Position 205 | 0.000 |
| Position 206 | 0.000 |
| Position 207 | 0.000 |
| Position 208 | 0.576 |
| Position 209 | 0.000 |
| Position 210 | 0.576 |
| Position 211 | 0.000 |
| Position 212 | 0.658 |
| Position 213 | 0.000 |
| Position 214 | 0.000 |
| Position 215 | 0.206 |
| Position 216 | 0.206 |
| Position 217 | 0.000 |
| Position 218 | 0.000 |
| Position 219 | 0.000 |
| Position 220 | 0.000 |
| Position 221 | 0.000 |
| Position 222 | 0.000 |
| Position 223 | 0.000 |
| Position 224 | 0.000 |
| Position 225 | 0.000 |
| Position 226 | 0.000 |
| Position 227 | 0.000 |
| Position 228 | 0.000 |
| Position 229 | 0.000 |
| Position 230 | 0.000 |
| Position 231 | 0.000 |
| Position 232 | 0.000 |
| Position 233 | 0.000 |
| Position 234 | 0.000 |
| Position 235 | 0.000 |
| Position 236 | 0.000 |
| Position 237 | 0.000 |
| Position 238 | 0.000 |
| Position 239 | 0.000 |
| Position 240 | 0.000 |
| Position 241 | 0.000 |
| Position 242 | 0.000 |
| Position 243 | 0.000 |
| Position 244 | 0.000 |
| Position 245 | 0.000 |
| Position 246 | 0.000 |
| Position 247 | 0.000 |
| Position 248 | 0.000 |
| Position 249 | 0.000 |
| Position 250 | 0.000 |
| Position 251 | 0.576 |
| Position 252 | 0.000 |
| Position 253 | 0.000 |
| Position 254 | 0.000 |
| Position 255 | 0.000 |
| Position 256 | 0.000 |
| Position 257 | 0.000 |
| Position 258 | 0.000 |
| Position 259 | 0.000 |
| Position 260 | 0.000 |
| Position 261 | 0.000 |
| Position 262 | 0.000 |
| Position 263 | 0.000 |
| Position 264 | 0.000 |
| Position 265 | 0.000 |
| Position 266 | 0.000 |
| Position 267 | 0.000 |
| Position 268 | 0.000 |
| Position 269 | 0.000 |
| Position 270 | 0.000 |
| Position 271 | 0.000 |
| Position 272 | 0.000 |
| Position 273 | 0.000 |
| Position 274 | 0.000 |
| Position 275 | 0.000 |
| Position 276 | 0.000 |
| Position 277 | 0.000 |
| Position 278 | 0.000 |
| Position 279 | 0.000 |
| Position 280 | 0.000 |
| Position 281 | 0.000 |
| Position 282 | 0.000 |
| Position 283 | 0.000 |
| Position 284 | 0.000 |
| Position 285 | 0.000 |
| Position 286 | 0.000 |
| Position 287 | 0.000 |
| Position 288 | 0.000 |
| Position 289 | 0.000 |
| Position 290 | 0.000 |
| Position 291 | 0.000 |
| Position 292 | 0.000 |
| Position 293 | 0.000 |
| Position 294 | 0.000 |
| Position 295 | 0.000 |
| Position 296 | 0.000 |
| Position 297 | 0.000 |
| Position 298 | 0.000 |
| Position 299 | 0.000 |
| Position 300 | 0.000 |
| Position 301 | 0.000 |
| Position 302 | 0.000 |
| Position 303 | 0.000 |
| Position 304 | 0.000 |
| Position 305 | 0.000 |
| Position 306 | 0.000 |
| Position 307 | 0.000 |
| Position 308 | 0.000 |
| Position 309 | 0.000 |
| Position 310 | 0.000 |
| Position 311 | 0.000 |
| Position 312 | 0.000 |
| Position 313 | 0.000 |
| Position 314 | 0.000 |
| Position 315 | 0.000 |
| Position 316 | 0.000 |
| Position 317 | 0.000 |
| Position 318 | 0.000 |
| Position 319 | 0.000 |
| Position 320 | 0.000 |
| Position 321 | 0.000 |
| Position 322 | 0.000 |
| Position 323 | 0.000 |
| Position 324 | 0.000 |
| Position 325 | 0.000 |
| Position 326 | 0.000 |
| Position 327 | 0.000 |
| Position 328 | 0.000 |
| Position 329 | 0.000 |
| Position 330 | 0.000 |
| Position 331 | 0.000 |
| Position 332 | 0.000 |
| Position 333 | 0.000 |
| Position 334 | 0.000 |
| Position 335 | 0.000 |
| Position 336 | 0.000 |
| Position 337 | 0.000 |
| Position 338 | 0.000 |
| Position 339 | 0.000 |
| Position 340 | 0.000 |
| Position 341 | 0.000 |
| Position 342 | 0.000 |
| Position 343 | 0.000 |
| Position 344 | 0.000 |
| Position 345 | 0.000 |
| Position 346 | 0.000 |
| Position 347 | 0.000 |
| Position 348 | 0.000 |
| Position 349 | 0.000 |
| Position 350 | 0.000 |
| Position 351 | 0.000 |
| Position 352 | 0.000 |
| Position 353 | 0.000 |
| Position 354 | 0.000 |
| Position 355 | 0.000 |
| Position 356 | 0.000 |
| Position 357 | 0.000 |
| Position 358 | 0.000 |
| Position 359 | 0.000 |
| Position 360 | 0.000 |
| Position 361 | 0.000 |
| Position 362 | 0.000 |
| Position 363 | 0.000 |
| Position 364 | 0.000 |
| Position 365 | 0.000 |
| Position 366 | 0.000 |
| Position 367 | 0.000 |
| Position 368 | 0.000 |
| Position 369 | 0.000 |
| Position 370 | 0.000 |
| Position 371 | 0.681 |
| Position 372 | 0.681 |
| Position 373 | 0.000 |
| Position 374 | 0.000 |
| Position 375 | 0.000 |
| Position 376 | 0.000 |
| Position 377 | 0.000 |
| Position 378 | 0.000 |
| Position 379 | 0.000 |
| Position 380 | 0.000 |
| Position 381 | 0.000 |
| Position 382 | 0.000 |
| Position 383 | 0.000 |
| Position 384 | 0.000 |
| Position 385 | 0.000 |
| Position 386 | 0.000 |
| Position 387 | 0.000 |
| Position 388 | 0.000 |
| Position 389 | 0.000 |
| Position 390 | 0.000 |
| Position 391 | 0.000 |
| Position 392 | 0.000 |
| Position 393 | 0.000 |
| Position 394 | 0.000 |
| Position 395 | 0.000 |
| Position 396 | 0.000 |
| Position 397 | 0.000 |
| Position 398 | 0.000 |
| Position 399 | 0.000 |
| Position 400 | 0.576 |
| Position 401 | 0.000 |
| Position 402 | 0.000 |
| Position 403 | 0.000 |
| Position 404 | 0.576 |
| Position 405 | 0.000 |
| Position 406 | 0.000 |
| Position 407 | 0.000 |
| Position 408 | 0.000 |
| Position 409 | 0.000 |
| Position 410 | 0.000 |
| Position 411 | 0.000 |
| Position 412 | 0.000 |
| Position 413 | 0.000 |
| Position 414 | 0.000 |
| Position 415 | 0.000 |
| Position 416 | 0.000 |
| Position 417 | 0.000 |
| Position 418 | 0.000 |
| Position 419 | 0.000 |
| Position 420 | 0.000 |
| Position 421 | 0.000 |
| Position 422 | 0.000 |
| Position 423 | 0.000 |
| Position 424 | 0.000 |
| Position 425 | 0.000 |
| Position 426 | 0.000 |
| Position 427 | 0.000 |
| Position 428 | 0.000 |
| Position 429 | 0.000 |
| Position 430 | 0.658 |
| Position 431 | 1.013 |
| Position 432 | 0.658 |
| Position 433 | 0.000 |
| Position 434 | 0.000 |
| Position 435 | 0.000 |
| Position 436 | 0.000 |
| Position 437 | 0.000 |
| Position 438 | 0.000 |
| Position 439 | 0.000 |
| Position 440 | 0.000 |
| Position 441 | 0.000 |
| Position 442 | 0.000 |
| Position 443 | 0.000 |
| Position 444 | 0.000 |
| Position 445 | 0.000 |
| Position 446 | 0.000 |
| Position 447 | 0.000 |
| Position 448 | 0.000 |
| Position 449 | 0.000 |
| Position 450 | 0.000 |
| Position 451 | 0.000 |
| Position 452 | 0.000 |
| Position 453 | 0.000 |
| Position 454 | 0.000 |
| Position 455 | 0.000 |
| Position 456 | 0.000 |
| Position 457 | 0.000 |
| Position 458 | 0.000 |
| Position 459 | 0.000 |
| Position 460 | 0.000 |
| Position 461 | 0.000 |
| Position 462 | 0.000 |
| Position 463 | 0.000 |
| Position 464 | 0.000 |
| Position 465 | 0.000 |
| Position 466 | 0.000 |
| Position 467 | 0.000 |
| Position 468 | 0.000 |
| Position 469 | 0.000 |
| Position 470 | 0.000 |
| Position 471 | 0.000 |
| Position 472 | 0.000 |
| Position 473 | 0.000 |
| Position 474 | 0.000 |
| Position 475 | 0.000 |
| Position 476 | 0.000 |
| Position 477 | 0.000 |
| Position 478 | 0.000 |
| Position 479 | 0.000 |
| Position 480 | 0.000 |
| Position 481 | 0.000 |
| Position 482 | 0.000 |
| Position 483 | 0.000 |
| Position 484 | 0.000 |
| Position 485 | 0.000 |
| Position 486 | 0.000 |
| Position 487 | 0.000 |
| Position 488 | 0.000 |
| Position 489 | 0.000 |
| Position 490 | 0.000 |
| Position 491 | 0.000 |
| Position 492 | 0.000 |
| Position 493 | 0.000 |
| Position 494 | 0.000 |
| Position 495 | 0.000 |
| Position 496 | 0.000 |
| Position 497 | 0.000 |
| Position 498 | 0.000 |
| Position 499 | 0.000 |
| Position 500 | 0.000 |
| Position 501 | 0.000 |
| Position 502 | 0.000 |
| Position 503 | 0.000 |
| Position 504 | 0.000 |
| Position 505 | 0.000 |
| Position 506 | 0.000 |
| Position 507 | 0.000 |
| Position 508 | 0.000 |
| Position 509 | 0.000 |
| Position 510 | 0.000 |
| Position 511 | 0.000 |
| Position 512 | 0.000 |
| Position 513 | 0.000 |
| Position 514 | 0.000 |
| Position 515 | 0.000 |
| Position 516 | 0.000 |
| Position 517 | 0.000 |
| Position 518 | 0.000 |
| Position 519 | 0.000 |
| Position 520 | 0.000 |
| Position 521 | 0.000 |
| Position 522 | 0.000 |
| Position 523 | 0.000 |
| Position 524 | 0.000 |
| Position 525 | 0.000 |
| Position 526 | 0.000 |
| Position 527 | 0.000 |
| Position 528 | 0.000 |
| Position 529 | 0.000 |
| Position 530 | 0.000 |
| Position 531 | 0.000 |
| Position 532 | 0.000 |
| Position 533 | 0.000 |
| Position 534 | 0.000 |
| Position 535 | 0.000 |
| Position 536 | 0.000 |
| Position 537 | 0.000 |
| Position 538 | 0.000 |
| Position 539 | 0.000 |
| Position 540 | 0.000 |
| Position 541 | 0.000 |
| Position 542 | 0.000 |
| Position 543 | 0.000 |
| Position 544 | 0.000 |
| Position 545 | 0.000 |
| Position 546 | 0.000 |
| Position 547 | 0.000 |
| Position 548 | 0.000 |
| Position 549 | 0.000 |
| Position 550 | 0.000 |
| Position 551 | 0.000 |
| Position 552 | 0.000 |
| Position 553 | 0.000 |
| Position 554 | 0.000 |
| Position 555 | 0.000 |
| Position 556 | 0.000 |
| Position 557 | 0.000 |
| Position 558 | 0.000 |
| Position 559 | 0.000 |
| Position 560 | 0.000 |
| Position 561 | 0.000 |
| Position 562 | 0.000 |
| Position 563 | 0.000 |
| Position 564 | 0.000 |
| Position 565 | 0.000 |
| Position 566 | 0.000 |
| Position 567 | 0.000 |
| Position 568 | 0.000 |
| Position 569 | 0.000 |
| Position 570 | 0.000 |
| Position 571 | 0.000 |
| Position 572 | 0.000 |
| Position 573 | 0.000 |
| Position 574 | 0.000 |
| Position 575 | 0.000 |
| Position 576 | 0.000 |
| Position 577 | 0.000 |
| Position 578 | 0.000 |
| Position 579 | 0.000 |
| Position 580 | 0.000 |
| Position 581 | 0.000 |
| Position 582 | 0.000 |
| Position 583 | 0.000 |
| Position 584 | 0.000 |
| Position 585 | 0.000 |
| Position 586 | 0.206 |
| Position 587 | 0.206 |
| Position 588 | 0.206 |
| Position 589 | 0.000 |
| Position 590 | 0.000 |
| Position 591 | 0.000 |
| Position 592 | 0.000 |
| Position 593 | 0.000 |
| Position 594 | 0.000 |
| Position 595 | 0.000 |
| Position 596 | 0.000 |
| Position 597 | 0.000 |
| Position 598 | 0.000 |
| Position 599 | 0.000 |
| Position 600 | 0.000 |
| Position 601 | 0.000 |
| Position 602 | 0.000 |
| Position 603 | 0.000 |
| Position 604 | 0.000 |
| Position 605 | 0.000 |
| Position 606 | 0.000 |
| Position 607 | 0.000 |
| Position 608 | 0.000 |
| Position 609 | 0.000 |
| Position 610 | 0.000 |
| Position 611 | 0.000 |
| Position 612 | 0.000 |
| Position 613 | 0.000 |
| Position 614 | 0.000 |
| Position 615 | 0.000 |
| Position 616 | 0.000 |
| Position 617 | 0.000 |
| Position 618 | 0.000 |
| Position 619 | 0.000 |
| Position 620 | 0.000 |
| Position 621 | 0.000 |
| Position 622 | 0.000 |
| Position 623 | 0.000 |
| Position 624 | 0.000 |
| Position 625 | 0.000 |
| Position 626 | 0.000 |
| Position 627 | 0.000 |
| Position 628 | 0.000 |
| Position 629 | 0.000 |
| Position 630 | 0.000 |
| Position 631 | 0.000 |
| Position 632 | 0.000 |
| Position 633 | 0.000 |
| Position 634 | 0.000 |
| Position 635 | 0.000 |
| Position 636 | 0.000 |
| Position 637 | 0.000 |
| Position 638 | 0.000 |
| Position 639 | 0.000 |
| Position 640 | 0.000 |
| Position 641 | 0.000 |
| Position 642 | 0.000 |
| Position 643 | 0.000 |
| Position 644 | 0.000 |
| Position 645 | 0.000 |
| Position 646 | 0.000 |
| Position 647 | 0.000 |
| Position 648 | 0.000 |
| Position 649 | 0.000 |
| Position 650 | 0.000 |
| Position 651 | 0.000 |
| Position 652 | 0.000 |
| Position 653 | 0.000 |
| Position 654 | 0.000 |
| Position 655 | 0.000 |
| Position 656 | 0.000 |
| Position 657 | 0.000 |
| Position 658 | 0.000 |
| Position 659 | 0.000 |
| Position 660 | 0.000 |
| Position 661 | 0.000 |
| Position 662 | 0.000 |
| Position 663 | 0.000 |
| Position 664 | 0.000 |
| Position 665 | 0.000 |
| Position 666 | 0.000 |
| Position 667 | 0.000 |
| Position 668 | 0.000 |
| Position 669 | 0.000 |
| Position 670 | 0.000 |
| Position 671 | 0.000 |
| Position 672 | 0.000 |
| Position 673 | 0.000 |
| Position 674 | 0.000 |
| Position 675 | 0.000 |
| Position 676 | 0.000 |
| Position 677 | 0.000 |
| Position 678 | 0.000 |
| Position 679 | 0.000 |
| Position 680 | 0.000 |
| Position 681 | 0.000 |
| Position 682 | 0.000 |
| Position 683 | 0.000 |
| Position 684 | 0.000 |
| Position 685 | 0.000 |
| Position 686 | 0.000 |
| Position 687 | 0.000 |
| Position 688 | 0.000 |
| Position 689 | 0.000 |
| Position 690 | 0.000 |
| Position 691 | 0.000 |
| Position 692 | 0.000 |
| Position 693 | 0.000 |
| Position 694 | 0.000 |
| Position 695 | 0.000 |
| Position 696 | 0.000 |
| Position 697 | 0.000 |
| Position 698 | 0.000 |
| Position 699 | 0.000 |
| Position 700 | 0.000 |
| Position 701 | 0.000 |
| Position 702 | 0.000 |
| Position 703 | 0.000 |
| Position 704 | 0.000 |
| Position 705 | 0.000 |
| Position 706 | 0.000 |
| Position 707 | 0.000 |
| Position 708 | 0.000 |
| Position 709 | 0.000 |
| Position 710 | 0.000 |
| Position 711 | 0.000 |
| Position 712 | 0.000 |
| Position 713 | 0.000 |
| Position 714 | 0.000 |
| Position 715 | 0.000 |
| Position 716 | 0.000 |
| Position 717 | 0.000 |
| Position 718 | 0.000 |
| Position 719 | 0.624 |
| Position 720 | 0.624 |
| Position 721 | 0.000 |
| Position 722 | 0.000 |
| Position 723 | 0.000 |
| Position 724 | 0.000 |
| Position 725 | 0.993 |
| Position 726 | 0.000 |
| Position 727 | 0.000 |
| Position 728 | 0.000 |
| Position 729 | 0.000 |
| Position 730 | 0.000 |
| Position 731 | 0.624 |
| Position 732 | 0.624 |
| Position 733 | 0.206 |
| Position 734 | 0.000 |
| Position 735 | 0.000 |
| Position 736 | 0.624 |
| Position 737 | 0.000 |
| Position 738 | 0.624 |
| Position 739 | 0.000 |
| Position 740 | 0.624 |
| Position 741 | 0.624 |
| Position 742 | 0.000 |
| Position 743 | 0.000 |
| Position 744 | 0.000 |
| Position 745 | 0.000 |
| Position 746 | 0.000 |
| Position 747 | 0.000 |
| Position 748 | 0.000 |
| Position 749 | 0.000 |
| Position 750 | 0.000 |
| Position 751 | 0.000 |
| Position 752 | 0.000 |
| Position 753 | 0.000 |
| Position 754 | 0.000 |
| Position 755 | 0.000 |
| Position 756 | 0.000 |
| Position 757 | 0.000 |
| Position 758 | 0.000 |
| Position 759 | 0.000 |
| Position 760 | 0.000 |
| Position 761 | 0.000 |
| Position 762 | 0.000 |
| Position 763 | 0.000 |
| Position 764 | 0.515 |
| Position 765 | 0.515 |
| Position 766 | 0.000 |
| Position 767 | 0.515 |
| Position 768 | 0.515 |
| Position 769 | 0.000 |
| Position 770 | 0.000 |
| Position 771 | 0.000 |
| Position 772 | 0.000 |
| Position 773 | 0.515 |
| Position 774 | 0.515 |
| Position 775 | 0.000 |
| Position 776 | 0.000 |
| Position 777 | 0.000 |
| Position 778 | 0.000 |
| Position 779 | 0.000 |
| Position 780 | 0.000 |
| Position 781 | 0.206 |
| Position 782 | 0.206 |
| Position 783 | 0.206 |

**Table S2:** *E. canis* gene p28 acid amin Entropy (*H* (x)). The *E. canis* strains selected for entropy comparison include samples from various countries, reflecting the geographical diversity of this species (Brazil: EF014897, MG584552, MG584553, MG584556; India: PP068999, PP068996, PP069000; Japan: LC844114; Thailand: OM337625; Vietnam: PQ659473 - PQ659482). Position based on the sequence of Brazil (EF014897).

| Position  acid amin | Entropy (H (x)) |
| --- | --- |
| Position 1 | 0.000 |
| Position 2 | 0.000 |
| Position 3 | 0.000 |
| Position 4 | 0.000 |
| Position 5 | 0.000 |
| Position 6 | 0.000 |
| Position 7 | 0.000 |
| Position 8 | 0.000 |
| Position 9 | 0.000 |
| Position 10 | 0.000 |
| Position 11 | 0.000 |
| Position 12 | 0.000 |
| Position 13 | 0.000 |
| Position 14 | 0.000 |
| Position 15 | 0.000 |
| Position 16 | 0.000 |
| Position 17 | 0.000 |
| Position 18 | 0.000 |
| Position 19 | 0.000 |
| Position 20 | 0.000 |
| Position 21 | 0.000 |
| Position 22 | 0.000 |
| Position 23 | 0.000 |
| Position 24 | 0.000 |
| Position 25 | 0.000 |
| Position 26 | 0.000 |
| Position 27 | 0.000 |
| Position 28 | 0.000 |
| Position 29 | 0.000 |
| Position 30 | 0.000 |
| Position 31 | 0.000 |
| Position 32 | 0.000 |
| Position 33 | 0.000 |
| Position 34 | 0.000 |
| Position 35 | 0.000 |
| Position 36 | 0.000 |
| Position 37 | 0.000 |
| Position 38 | 0.000 |
| Position 39 | 0.000 |
| Position 40 | 0.000 |
| Position 41 | 0.000 |
| Position 42 | 0.000 |
| Position 43 | 0.000 |
| Position 44 | 0.000 |
| Position 45 | 0.000 |
| Position 46 | 0.436 |
| Position 47 | 0.658 |
| Position 48 | 0.633 |
| Position 49 | 0.000 |
| Position 50 | 0.000 |
| Position 51 | 0.000 |
| Position 52 | 0.000 |
| Position 53 | 0.000 |
| Position 54 | 0.000 |
| Position 55 | 0.000 |
| Position 56 | 0.000 |
| Position 57 | 0.000 |
| Position 58 | 0.000 |
| Position 59 | 0.000 |
| Position 60 | 0.766 |
| Position 61 | 0.000 |
| Position 62 | 0.000 |
| Position 63 | 0.000 |
| Position 64 | 0.000 |
| Position 65 | 0.000 |
| Position 66 | 0.000 |
| Position 67 | 0.000 |
| Position 68 | 0.515 |
| Position 69 | 0.000 |
| Position 70 | 0.576 |
| Position 71 | 0.658 |
| Position 72 | 0.206 |
| Position 73 | 0.000 |
| Position 74 | 0.000 |
| Position 75 | 0.000 |
| Position 76 | 0.000 |
| Position 77 | 0.000 |
| Position 78 | 0.000 |
| Position 79 | 0.000 |
| Position 80 | 0.000 |
| Position 81 | 0.000 |
| Position 82 | 0.000 |
| Position 83 | 0.000 |
| Position 84 | 0.576 |
| Position 85 | 0.000 |
| Position 86 | 0.000 |
| Position 87 | 0.000 |
| Position 88 | 0.000 |
| Position 89 | 0.000 |
| Position 90 | 0.000 |
| Position 91 | 0.000 |
| Position 92 | 0.000 |
| Position 93 | 0.000 |
| Position 94 | 0.000 |
| Position 95 | 0.000 |
| Position 96 | 0.000 |
| Position 97 | 0.000 |
| Position 98 | 0.000 |
| Position 99 | 0.000 |
| Position 100 | 0.000 |
| Position 101 | 0.000 |
| Position 102 | 0.000 |
| Position 103 | 0.000 |
| Position 104 | 0.000 |
| Position 105 | 0.000 |
| Position 106 | 0.000 |
| Position 107 | 0.000 |
| Position 108 | 0.000 |
| Position 109 | 0.000 |
| Position 110 | 0.000 |
| Position 111 | 0.000 |
| Position 112 | 0.000 |
| Position 113 | 0.000 |
| Position 114 | 0.000 |
| Position 115 | 0.000 |
| Position 116 | 0.000 |
| Position 117 | 0.000 |
| Position 118 | 0.000 |
| Position 119 | 0.000 |
| Position 120 | 0.000 |
| Position 121 | 0.000 |
| Position 122 | 0.000 |
| Position 123 | 0.000 |
| Position 124 | 0.681 |
| Position 125 | 0.000 |
| Position 126 | 0.000 |
| Position 127 | 0.000 |
| Position 128 | 0.000 |
| Position 129 | 0.000 |
| Position 130 | 0.000 |
| Position 131 | 0.000 |
| Position 132 | 0.000 |
| Position 133 | 0.000 |
| Position 134 | 0.576 |
| Position 135 | 0.576 |
| Position 136 | 0.000 |
| Position 137 | 0.000 |
| Position 138 | 0.000 |
| Position 139 | 0.000 |
| Position 140 | 0.000 |
| Position 141 | 0.000 |
| Position 142 | 0.000 |
| Position 143 | 0.000 |
| Position 144 | 1.264 |
| Position 145 | 0.000 |
| Position 146 | 0.000 |
| Position 147 | 0.000 |
| Position 148 | 0.000 |
| Position 149 | 0.000 |
| Position 150 | 0.000 |
| Position 151 | 0.000 |
| Position 152 | 0.000 |
| Position 153 | 0.000 |
| Position 154 | 0.000 |
| Position 155 | 0.000 |
| Position 156 | 0.000 |
| Position 157 | 0.000 |
| Position 158 | 0.000 |
| Position 159 | 0.000 |
| Position 160 | 0.000 |
| Position 161 | 0.000 |
| Position 162 | 0.000 |
| Position 163 | 0.000 |
| Position 164 | 0.000 |
| Position 165 | 0.000 |
| Position 166 | 0.000 |
| Position 167 | 0.000 |
| Position 168 | 0.000 |
| Position 169 | 0.000 |
| Position 170 | 0.000 |
| Position 171 | 0.000 |
| Position 172 | 0.000 |
| Position 173 | 0.000 |
| Position 174 | 0.000 |
| Position 175 | 0.000 |
| Position 176 | 0.000 |
| Position 177 | 0.000 |
| Position 178 | 0.000 |
| Position 179 | 0.000 |
| Position 180 | 0.000 |
| Position 181 | 0.000 |
| Position 182 | 0.000 |
| Position 183 | 0.000 |
| Position 184 | 0.000 |
| Position 185 | 0.000 |
| Position 186 | 0.000 |
| Position 187 | 0.000 |
| Position 188 | 0.000 |
| Position 189 | 0.000 |
| Position 190 | 0.000 |
| Position 191 | 0.000 |
| Position 192 | 0.000 |
| Position 193 | 0.000 |
| Position 194 | 0.000 |
| Position 195 | 0.000 |
| Position 196 | 0.206 |
| Position 197 | 0.000 |
| Position 198 | 0.000 |
| Position 199 | 0.000 |
| Position 200 | 0.000 |
| Position 201 | 0.000 |
| Position 202 | 0.000 |
| Position 203 | 0.000 |
| Position 204 | 0.000 |
| Position 205 | 0.000 |
| Position 206 | 0.000 |
| Position 207 | 0.000 |
| Position 208 | 0.000 |
| Position 209 | 0.000 |
| Position 210 | 0.000 |
| Position 211 | 0.000 |
| Position 212 | 0.000 |
| Position 213 | 0.000 |
| Position 214 | 0.000 |
| Position 215 | 0.000 |
| Position 216 | 0.000 |
| Position 217 | 0.000 |
| Position 218 | 0.000 |
| Position 219 | 0.000 |
| Position 220 | 0.000 |
| Position 221 | 0.000 |
| Position 222 | 0.000 |
| Position 223 | 0.000 |
| Position 224 | 0.000 |
| Position 225 | 0.000 |
| Position 226 | 0.000 |
| Position 227 | 0.000 |
| Position 228 | 0.000 |
| Position 229 | 0.000 |
| Position 230 | 0.000 |
| Position 231 | 0.000 |
| Position 232 | 0.000 |
| Position 233 | 0.000 |
| Position 234 | 0.000 |
| Position 235 | 0.000 |
| Position 236 | 0.000 |
| Position 237 | 0.000 |
| Position 238 | 0.000 |
| Position 239 | 0.000 |
| Position 240 | 0.624 |
| Position 241 | 0.000 |
| Position 242 | 0.993 |
| Position 243 | 0.000 |
| Position 244 | 0.624 |
| Position 245 | 0.206 |
| Position 246 | 0.624 |
| Position 247 | 0.624 |
| Position 248 | 0.000 |
| Position 249 | 0.000 |
| Position 250 | 0.000 |
| Position 251 | 0.000 |
| Position 252 | 0.000 |
| Position 253 | 0.000 |
| Position 254 | 0.000 |
| Position 255 | 0.515 |
| Position 256 | 0.515 |
| Position 257 | 0.000 |
| Position 258 | 0.515 |
| Position 259 | 0.000 |
| Position 260 | 0.000 |
| Position 261 | 0.206 |
